# Influence of PECAM‐1 ligand interactions on PECAM‐1‐dependent cell motility and filopodia extension

**DOI:** 10.14814/phy2.13030

**Published:** 2016-11-28

**Authors:** Valsamma Abraham, Andrew Parambath, Debria S. Joe, Horace M. DeLisser

**Affiliations:** ^1^ Pulmonary, Allergy and Critical Care DivisionDepartment of MedicinePerelman School of School of MedicineUniversity of PennsylvaniaPhiladelphiaPennsylvania

**Keywords:** Cdc42, endothelial cells, filopodia, motility, PECAM‐1

## Abstract

Platelet endothelial cell adhesion molecule (PECAM‐1) has been implicated in angiogenesis through processes that involve stimulation of endothelial cell motility. Previous studies suggest that PECAM‐1 tyrosine phosphorylation mediates the recruitment and then activation of the tyrosine phosphatase SHP‐2, which in turn promotes the turnover of focal adhesions and the extension of filopodia, processes critical to cell motility. While these studies have implicated PECAM‐1‐dependent signaling in PECAM‐1‐mediated cell motility, the involvement of PECAM‐1 ligand binding in cell migration is undefined. Therefore to investigate the role of PECAM‐1 binding interactions in cell motility, mutants of PECAM‐1 were generated in which either homophilic or heparin/glycosaminoglycan (GAG)‐mediated heterophilic binding had been disabled and then expressed in an endothelial cell surrogate. We found that the ability of PECAM‐1 to stimulate cell migration, promote filopodia formation and trigger Cdc42 activation were lost if PECAM‐1‐dependent homophilic or heparin/GAG‐dependent heterophilic ligand binding was disabled. We further observed that PECAM‐1 concentrated at the tips of extended filopodia, an activity that was diminished if homophilic, but not heparin/GAG‐mediated heterophilic binding had been disrupted. Similar patterns of activities were seen in mouse endothelial cells treated with antibodies that specifically block PECAM‐1‐dependent homophilic or heterophilic adhesion. Together these data provide evidence for the differential involvement of PECAM‐1‐ligand interactions in PECAM‐1‐dependent motility and the extension of filopodia.

## Introduction

Platelet endothelial cell adhesion molecule‐1 (PECAM‐1), expressed on the endothelium, has been implicated in in vivo angiogenesis (DeLisser et al. 1997; Solowiej et al. 2003; Cao et al. 2009), including tumor angiogenesis (Zhou et al. 1999; Cao et al. 2002, 2009) and in postnatal retinal and lung vascular development (DeLisser et al. 2006; Dimaio et al. 2008). Its activity in the process of angiogenesis appears to involve the stimulation or enhancement of endothelial cell (EC) motility (Cao et al. 2002, 2009; Gratzinger et al. 2003; O'Brien et al. 2004; Kondo et al. 2007; Zhu et al. 2010). At least two mechanisms have been identified by which PECAM‐1 promotes EC migration. First, PECAM‐1 stimulates the turnover of focal adhesions of migrating cells (O'Brien et al. 2004), phenomena that appear to be dependent on PECAM‐1 tyrosine phosphorylation of its cytoplasmic domain (Jackson et al. 1997; Lu et al. 1997; Sagawa et al. 1997; Cao et al. 1998; Kogata et al. 2003) and the subsequent PECAM‐1‐mediated recruitment to the cell membrane and then activation of the SHP‐2 phosphatase (Zhu et al. 2010). In addition, PECAM‐1 promotes the formation of filopodia through processes that may involve SHP‐2 mediated activation of ERK as well as others that act to increase the expression of Cdc42 (Zhu et al. 2010).

The various effects of PECAM‐1 on cell function are mediated by the binding of PECAM‐1 to other PECAM‐1 molecules (homophilic binding) or to non‐PECAM‐1 molecules (heterophilic binding), such as heparin/heparan sulfate glycosaminoglycan (GAG)‐containing proteoglycans (DeLisser et al. 1993, 1994; Yan et al. 1995; Sun et al. 1996a, 1996b; Famiglietti et al. 1997). Structurally, the extracellular domain is composed of six Ig‐like domains (Woodfin et al. 2007), with distinct residues in Ig‐like domain 1 (Newton et al. 1997; Nakada et al. 2000) and Ig‐like domains 2 and 3 (Coombe et al. 2008), respectively, mediating homophilic and heparin/GAG‐dependent heterophilic binding. For homophilic interactions, molecules on adjacent cells overlap each other in an antiparallel manner in which Ig‐like domain 1 of one molecule binds to Ig‐like domain 2 of the counter molecule (Sun et al. 1996a, 1996b). In terms of factors that regulate the specificity of ligand interaction, a high surface density, antibody‐mediated engagement of the membrane proximal Ig‐like domain 6 and the absence of sequences from the cytoplasmic domain coded by exon 14 of the PECAM‐1 gene promote homophilic over GAG‐dependent heterophilic adhesion (Sun et al. 1996a, 1996b). In addition, homophilic adhesion is dependent on the glycosylation state of the PECAM‐1 molecule (Lee et al. 2014).

While the recruitment and activation of SHP‐2 by PECAM‐1 have implicated a role for PECAM‐1‐dependent signaling in PECAM‐1‐mediated cell motility (O'Brien et al. 2004; Zhu et al. 2010), the involvement of PECAM‐1 ligand binding in cell migration has not been well defined (Cao et al. 2009). Therefore, to assess the role of PECAM‐1 binding interactions in cell motility, mutants of PECAM‐1 were generated in which either homophilic or heparin/GAG‐mediated heterophilic binding had been disabled and then expressed in an EC surrogate. We found that the ability of PECAM‐1 to stimulate cell migration, promote filopodia formation and trigger Cdc42 activation were lost if PECAM‐1‐dependent homophilic or heparin/GAG‐dependent heterophilic ligand binding was disabled. We further observed that PECAM‐1 concentrated at the tips of extended filopodia, an activity that diminished if homophilic, but not heparin/GAG‐mediated heterophilic binding had been lost. Similar patterns of activities were seen in mouse ECs treated with anti‐mouse PECAM‐1 antibodies that specifically blocked PECAM‐1‐dependent homophilic or heterophilic adhesion. Together these data support the differential involvement of PECAM‐1‐ligand interactions in PECAM‐1‐dependent motility and the extension of filopodia.

## Materials and Methods

### Chemicals and reagents

All chemicals, reagents, and primers were obtained from Sigma (St. Louis, MO) unless otherwise specified. Restriction enzymes, *Taq* DNA polymerase, and Phusion high fidelity DNA polymerase were purchased from New England BioLabs, Inc. (Beverly, MA). Heparin Cy5.5 was obtained from Nanocs Inc, (New York, NY). 7‐amino‐actinomycin D (7AAD) was obtained from BD Transduction Laboratories (Lexington, KY).

### Antibodies

The following antibodies against human proteins were employed unless otherwise noted: goat (M20) and rabbit (M185) polyclonal anti‐mouse PECAM‐1 antibodies and anti‐GAPDH antibody from Santa Cruz Biotechnology (Santa Cruz, CA); 390, rat anti‐mouse PECAM‐1 antibody (DeLisser et al. 1997), MEC 13.3, rat anti‐mouse PECAM‐1 (DeLisser et al. 1997) and DyLight650 conjugated antibody from Novus Biologicals (Littleton CO); anti‐mouse CD31, Alexa 647 conjugated antibody from Southern Biotech (Birmingham, AL); 390, MEC 13.3 and rat IgG2a, *κ* isotype control from BioLegend (San Diego, CA); donkey anti‐goat IgG, goat anti‐mouse alexa594 conjugated from Life Technologies (Grand Island, NY); anti‐paxillin antibody (BD Transduction Laboratories (Lexington, KY); antiphosphotyrosine antibody and HRP‐conjugated, goat anti‐mouse antibody from EMD Millipore (Billerica, MA); and anti‐EGFR and anti‐Cdc42 antibodies from Cell Signaling Technology (Danvers, MA).

### Cell lines

Human embryonic kidney (HEK) 293T cells and the H5V murine endothelial cells (Garlanda et al. 1994) were maintained in Dulbecco's Modified Eagle's Medium (DMEM) containing 1.0 g/L glucose, 2 mmol/L l‐glutamine, 100 U/mL penicillin, 0.1 μg/mL streptomycin and 10% fetal bovine serum (FBS). REN cells (a human mesothelioma cell line) (Smythe et al. 1994) were grown in RPMI1640 with 2 mmol/L l‐glutamine, 100U/mL penicillin, 0.1   μg/mL streptomycin, and 10% FBS. Stable transduced REN cell lines expressing WT and mutant PECAM‐1 were cultured in RPMI 1640 complete media with 1 μg/mL puromycin. Primary murine endothelial cells were isolated as previously described (Fehrenbach et al. 2009) and cultured in M199 medium containing 15% FBS, 50 μg/mL endothelial growth factor (BD Bioscience, San Jose, CA), 100 μg/mL heparin and 1 mmol/L glutamine. Cells were regularly passaged two times week to maintain them under exponential growth conditions.

### Generation of lentiviral vector constructs expressing the wild‐type or mutant murine PECAM‐1 cDNA

Full‐length murine PECAM‐1 and its mutants were expressed in the lentiviral cDNA expression vector, pCDH‐CMV‐MCS‐EF1‐GFP‐Puro (System Biosciences, Mountain View, CA) as described below. The full‐length cDNA of murine PECAM‐1 was excised from the pcDNAI/Neo vector (Sun et al. 2000) and the insert subcloned into the Not I restriction sites of the expression vector pcDNA3.1(+) (Invitrogen, Carlsbad, CA) using the In‐Fusion^™^ Advantage PCR Cloning Kit from Clontech Laboratories (Mountain View, CA). The resulting vector, designated pCDNA3‐MP, was then used as a backbone to generate mutants, by site‐directed mutagenesis, in which homophilic binding (pCDNA3‐MP_ΔHom_), heterophilic binding (pCDNA3‐MP_ΔHet_), or PECAM‐1 tyrosine phosphorylation (pCDNA3‐ MP_Y→F_) had been eliminated using the Quick Change Lightening Mutagenesis Kit from Agilent Technologies (Santa Clara, CA). (The primers used to generate the mutations are available upon request). PECAM‐1 cDNA were then PCR amplified from the various pCDNA3‐MP vectors. The sequences of the primer pair used to generate the full‐length mouse PECAM‐1 were as follows: 5′AGATTCTAGA*GCTAGC* ATGCTCCTGGCTCTGGGACTC‐3′(pCDH PECAM‐1 FL forward primer) and 5′‐CAGATCCTT*GCGGCCGC* TTAAGTTCCATTAAGGGAGCCTTC‐3′ (pCDH PECAM‐1 reverse primer), with Nhe1 and Not 1 sequences in italics. In addition to the gene‐specific sequences, the sequences of the primer pair forward and reverse contained about 16 bp extensions (from both 5′ and 3′ ends) that are homologs to the ends of the destination vector. The PCR‐amplified PECAM‐1 CDNA was cloned into Nhe1 and Not I site of the PCDH lentiviral vector using the In‐Fusions ^™^ Advantage PCR Cloning Kit (Clontech, Mountain View, CA). The DNA sequences of the constructs were confirmed by sequencing.

### Vector production and concentration

3 × 10^6^ 293T cells were seeded in 10 cm tissue dishes 24 h prior to transfection. Cell culture media was replaced with 9 mL of fresh media containing no antibiotics 2 h before transfection. Transfection was carried out according to the manufacturer's instructions (System Bioscience, Palo Alto, CA). Briefly 293T cells were cotransfected with 20 μL pPACK H1 packaging plasmid mix and 2 μg of lentiviral pCDH PECAM‐1 vector per each 10 cm dish using pure Fection as a transfection reagent. Twenty‐four hours after initiating transfection, the plasmid – pureFection solution was removed, and replaced with complete medium. Cells were cultured for another 24–48 h. Lentivirus‐containing supernatants were collected at 48 and 72 h after transfection and centrifuged at 3000 × *g* for 15 min at room temperature to pellet cell debris. The viral particles were concentrated with PEG‐it virus precipitation solution. The viral pellet was resuspended in sterile PBS at 1/100 of the original volume. The viral stock was aliquoted in cryogenic vials and stored at −80°C until ready for use. After transfection, the viral titer was determined by counting GFP‐positive cells by fluorescence microscopy. 293T cells were plated at 5 × 10^4^ cells/well in a 24 well plate in 1 mL DMEM containing 10% serum, l‐glutamine, and antibiotics. Twenty‐four hours later, cells in each well were transduced with 5 fold dilutions of vector encoding GFP. Forty‐eight hours after transduction cells were analyzed for GFP expression. Transducing units/mL was calculated as follows: number of GFP‐positive colonies counted × dilution factor × 40.

### Transduction of REN cells

One day prior to transduction, REN cells were plated in 24‐well plates at 5 × 10^4^ cells. After 24 h, REN cells were infected with lentiviral particles containing full‐length murine PECAM‐1 cDNA or variants of PECAM‐1. After 72 h. The cells were grown in selective (puromycin 1.5 μg/mL) for 2 weeks and subsequently (1.0 μg/mL), in order to establish stably transfected REN cells expressing mouse PECAM‐1. After 14 days the cells were stained with murine PECAM‐1 Ab (mAb 390) and the cells expressing murine PECAM‐1 sorted using a BD FACS Aria II SORP, from BD Biosciences (San Jose, CA). The expanded cells were used for further experiments. PECAM‐1 expression was confirmed by immunoblotting and fluorescence‐activated cell sorting (FACS) analysis.

### Immunoprecipitation and Western blotting

The various cell lines were cultured to confluence and then cultured for 16 h with media containing 1% serum. The cells were either treated or untreated with 0.5 mmol/L pervandate solution for 2 h. The cells were mechanically stimulated by wounding the confluent monolayers. After 3 h the cells were then washed with cold PBS and lysed in 400 μL of lysis buffer [20 mmol/L Tris‐HCl (pH 7.5), 150 mmol/L NaCl, 1 mmol/L Na2EDTA, 1 mmol/L EGTA, 1% Triton, 2.5 mmol/L sodium pyrophosphate, 1 mmol/L β‐glycerophosphate, 1 mmol/L Na3VO4, 1 μg/mL leupeptin, and 1 mmol/L PMSF) for 20 min on ice. The resulting protein extracts were sonicated briefly and protein concentration was determined by BCA assay. The lysates were precleared with protein G agarose (Santa Cruz) for 30 min at 4°C and immunoprecipitated with 2 μg of goat anti‐murine PECAM‐1 polyclonal antibody overnight at 4°C. Protein G agarose beads were added and incubated for an additional 2 h. After immunoprecipitation the beads were washed four times with lysis buffer. The proteins were then separated on 4–12% Bis Tris gel (Invitrogen) and transferred to nitrocellulose membrane. Membranes were blocked for 1 h in 4% BSA‐PBS Tween 20 and incubated in antiphosphotyrosine antibody overnight (1:2000 dilution), washed for 20 min and then incubated in HRP‐conjugated Goat anti‐mouse antibody. After washing, the blots were then developed with ECL (Amersham, Pharmacia Biotech, Piscataway, NJ) according to manufacturer's instructions.

Total proteins were extracted by RIPA lysis buffer and protein concentration was determined by BCA protein assay (Thermo Scientific Peirce, Rockford, IL). Proteins were then separated by 10% SDS‐PAGE and transferred to PVDF membranes (Millipore), incubated in primary antibodies (1:1000) overnight at 4°C and then in HRP‐conjugated secondary antibodies (1:10,000).

### Fluorescent activated cell sorting (FACS) analysis

REN cell lines were detached from tissue culture plates by enzyme free cell dissociation buffer (Life Technologies), washed with PBS and resupended in PBS with 2% BSA. The cells were incubated with fluorescently tagged anti‐PECAM‐1 antibody on ice for 30 min. Cells were then washed twice with PBS with 2% BSA. FACS was performed on LSR 11 Flow Cytometer (BD Bioscience) and the data analyzed with Flowjo software (Tree Star, Ashland OR). For the assessment of heparin binding, 1 × 10^6^ cells were stained with heparin Cy5.5 (4 μg) for 45 min on ice and washed twice with PBS with 2% BSA and analyzed by flow cytometry. 7‐amino‐actinomycin D (7AAD) was used to identify nonviable cells.

### Immunofluorescence staining

Transduced REN cells were plated on four‐chamber polystyrene vessel glass slides (BD Falcon, Bedford, MA) at a density of 20 000/well. The confluent cells were washed once with PBS and fixed in 4% paraformaldehyde (Polysciences, Warrington, PA) for 15 min at room temperature. The cells were incubated in PBS with 0.3% Triton X‐100, 5% normal rabbit serum, and 1% bovine serum albumin for 60 min at room temperature to permeabilize the plasma membrane and block nonspecific‐binding sites. The cells were incubated overnight at 4°C with goat anti‐mouse PECAM‐1 polyclonal antibody (1:200 dilution). After incubation, the cells were washed three times with PBS and then incubated 1 h at room temperature with donkey, anti‐goat IgG, alexa594 conjugated (1:500 dilution). Counterstaining was done using Hoechst (blue) and mounted on glass slide with Aqua‐Polymount (Polysciences). Images were captured with a Leica TCS SP5II scanning laser confocal microscope, using 405 nm and 594 nm lasers and a 63 × objective (5–6 μmol/L stacks, 0.13 μm steps). For single cell staining for the localization of PECAM‐1 in the tips of filopodia, procedures were as described above, except that 10 000 cells/well were plated. For paxillin staining, the confluent monolayer was wounded. After wounding the cells were grown an additional 24 h in media containing 1% serum. After blocking for 1 h, cells were stained with anti‐paxillin antibody (1:100 dilution) overnight, followed by incubation with Alexa Fluor 594 goat anti‐mouse IgG.

### Mixed aggregation assay

The aggregation assay used in these studies was adapted from previously published procedures (DeLisser et al. 1993). The various REN cell lines were nonenzymatically detached from the tissue culture dish and resuspended in complete RPMI medium. The cells were then washed twice with Hanks Balance Salt Solution (HBSS) without any divalent cations and cells were then resuspended in HBSS solution to a concentration of 200 000 cells/mL. To determine whether aggregation involved homophilic or heterophilic adhesion, 0.5 mL of control REN cells (non‐GFP‐expressing) were mixed with 0.5 mL of REN‐MP, REN‐MP_ΔHom_, or REN‐MP_ΔHet_ (all GFP‐expressing) in a 24‐well plate, which had been precoated with 2% BSA for 3 h. The plate was then incubated at 37°C for 10 min with gentle agitation using a rotating plate at 70 rpm. The cells were subsequently examined under UV light with fluorescein filters or under phase contrast using Nikon EclipseTE 2000 microscope, to, respectively, identify the PECAM‐1‐expressing REN cells versus the control REN cells. The number of fluorescent and nonfluorescent cells in each aggregate of 5 and 6 cells were counted. Quantitative analysis was performed as previously described (DeLisser et al. 1993, 1994; Sun et al. 1996a). Specifically for the 5 cell aggregates, aggregates were defined as homophilic if they contained 4 or 5 PECAM‐1‐expressing REN cells; heterophilic if 2 or 3 PECAM‐1‐expressing cells were present; or nonspecific if the aggregate was composed of one or no PECAM‐1‐expressing cell. For the 6 cell aggregates, for which there were seven types of aggregates, aggregates were defined as homophilic if they contained 6 PECAM‐1‐expression cells; heterophilic if the aggregate was composed of 3 PECAM‐1‐expressing and 3 REN cells; and nonspecific if only REN cells were present. This was done first because of the uncertainty in categorizing aggregates with 4 PECAM‐1‐expressing and 2 REN cells as homophilic or heterophilic, or the aggregates with 2 PECAM‐1‐expressing and 4 REN cells as heterophilic or nonspecific, and second to provide for equal numbers in the comparisons of the aggregate types. For the mixed aggregation studies using purified anti‐mouse PECAM‐1 antibodies 390 and Mec13, the procedures were as described above except the cells were incubated in the presence of 50 μg/mL of IgG or antibody for 30 min at 37°C. Data were then expressed as the ratio of homophilic to heterophilic aggregates. For each experiment at least 25 aggregates were identified for the analyses.

### In vitro wound‐induced migration assay

The wounding of confluent cell monolayers was modified from previously published procedures (DeLisser et al. 1993; O'Brien et al. 2004). Twenty thousand cells were added to 24‐well tissue culture plates and allowed to grow to confluence. Linear defects were then made in the monolayer. The wounded culture was washed with PBS and then incubated for 24 h in media containing 1% serum. Using computer‐assisted image analysis and the Image‐Pro Plus software (Media Cybernetics, Rockville, MD), images were obtained immediately after wounding, and then 24 h later, and the change in wound area was determined. For each cell type 3–5 wounds were analyzed for each experiment.

### Cdc42 activation assay

Cdc42 activity was measured according to the manufacturer's instructions (Millipore). Briefly, cell lysates were prepared from subconfluent (80–90%) cultured cells. Cells were washed twice with ice‐cold PBS, and lysed with lysis buffer containing 25 mm HEPES, pH 7.5, 150 mmol/L NaCl, 1% Igepal CA‐630, 10 mmol/L MgCl_2_, 1 mmol/L EDTA, 10% glycerol, 1 mmol/L Na_3_VO_4_, 25 mmol/L NaF, 10 μg/mL aprotinin, and 10 μg/mL leupeptin, for 20 min at 4°C. The lysates were sonicated three times for 10 sec and insoluble materials were removed by centrifugation. Next, 10 μg of PAK‐1PBD‐agarose beads, which specifically binds to active Cdc42 were added to the cell lysates and incubated for 90 min at 4°C with gentle agitation. The agarose beads were washed three times with lysis buffer and boiled in 2× Laemmli reducing sample buffer. Samples were resolved by SDS‐PAGE and immunoblotted with anti‐Cdc42 antibody.

### Statistical analysis

Statistical analyses were carried out using GraphPad Prism 5 software (version 5.01; GraphPad Software, Inc., CA, USA) and one‐way analysis of variance (ANOVA) using Bonferroni correction was used for multiple comparisons.

## Results

### Expression of murine PECAM‐1 constructs in REN cells

The expression of PECAM‐1 in the REN cells (a mesothelioma cell line) (Smythe et al. 1994) has served as an important in vitro system for studying the functions of endothelial PECAM‐1 (Muzykantov et al. 1999; Nakada et al. 2000; Sun et al. 2000; Cao et al. 2002, 2009; Ji et al. 2002; Wiewrodt et al. 2002; O'Brien et al. 2004; Garnacho et al. 2008; Zhu et al. 2010; Chacko et al. 2012). Although of tumor cell origin, REN cells have several features that have made them an appealing system for modeling the vascular endothelium. These include the fact that like ECs, REN cells form cobblestone cell monolayers, and while they lack PECAM‐1, REN cells express several relevant endothelial surface molecules (e.g., αvβ3, ICAM‐1, VCAM‐1, and VEGFR‐1). Furthermore, for both ECs and REN cell transduced to express PECAM‐1 (1) PECAM‐1 concentrates at cell–cell junctions (Sun et al. 2000; O'Brien et al. 2004); (2) tube‐like structures form on Matrigel (Cao et al. 2002; O'Brien et al. 2004; Zhu et al. 2010); (3) H_2_O_2_ activates a calcium‐permeant, nonselective cation current (Ji et al. 2002); (4) the internalization and intercellular trafficking of surface bound anti‐PECAM‐1 antibodies are entirely comparable (Muzykantov et al. 1999; Wiewrodt et al. 2002; Garnacho et al. 2008; Chacko et al. 2012); and (5) wound‐induced cell migration is associated with PECAM‐1 tyrosine phosphorylation and SHP‐2 association (O'Brien et al. 2004; Zhu et al. 2010). Most importantly, with respect to endothelial cell motility, filopodia formation and Cdc42 levels, the issues addressed in this paper, PECAM‐1‐expressing REN cell when compared with control REN cells, replicate the behavior of wild‐type versus PECAM‐1‐null murine ECs (Cao et al. 2009).

**Figure 1:**
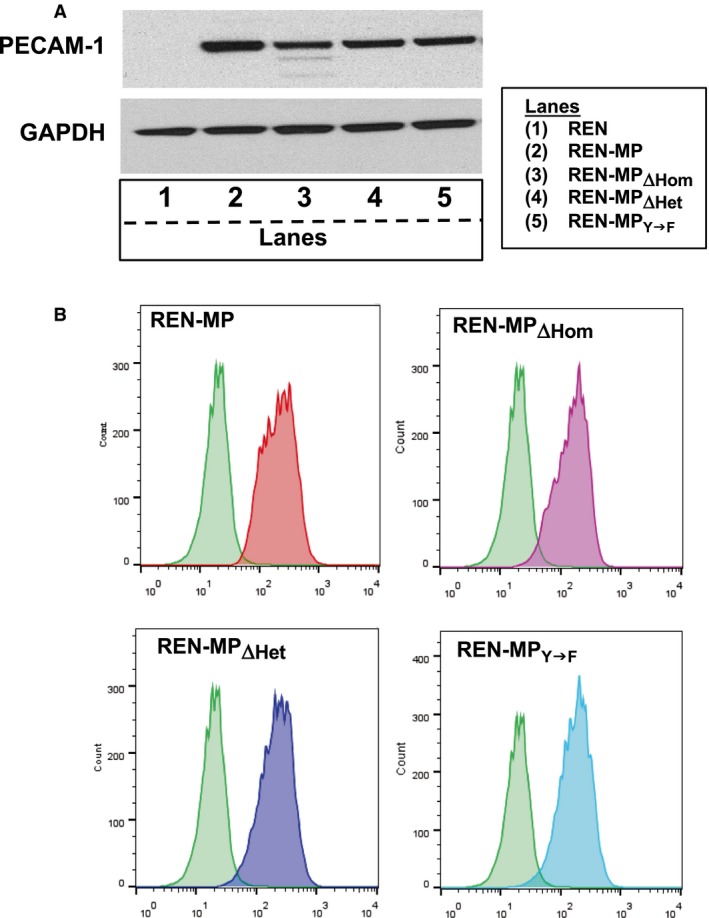
Expression of constructs of mouse PECAM‐1 in REN cells. (A) Cell lysates from REN, REN‐MP, REN‐MP_ΔHom_, REN‐MP_ΔHet, and_ REN‐MP_Y→F_ cell lines were immunoblotted with anti‐PECAM‐1 antibody. (B) REN, REN‐MP, REN‐MP_ΔHom_, REN‐MP_ΔHet_, and REN‐MP_Y→F_ cells were stained with anti‐PECAM‐1 antibody and then subjected to Fluorescence‐activated cell sorting (FACS) analysis. The green tracing in each panel represents background staining with control isotype IgG. Both approaches confirmed high and comparable levels of PECAM‐1 in the transduced cells.

To investigate the involvement of PECAM‐1 ligand interactions in PECAM‐1‐dependent cell motility a series of PECAM‐1‐expressing REN cell lines were generated. To accomplish this, REN cells were transduced with lentiviral constructs expressing either wild‐type mouse PECAM‐1 (REN‐MP), or mouse PECAM‐1 bearing mutations that disabled homophilic binding (REN‐MP_ΔHom_), heparin/GAG‐mediated heterophilic binding (REN‐MP_ΔHet_) or tyrosine phosphorylation of the cytoplasmic domain (REN‐MP_Y→F_) (Table 1). Western blot and FACS analyses confirmed the expression of these constructs to be at comparable levels on the surface of the REN cells (Fig. 1).

**Table 1:** REN cells expressing wild‐type or mutant mouse PECAM‐1

REN cell type	Description	Mutations
REN‐MP	Wild‐type mouse PECAM‐1	N/A
REN‐MP_ΔHom_	PECAM‐1 homophilic binding eliminated	H11, D33, K50, D51 and K89 from domain 1, all mutated to alanine
REN‐MP_ΔHet_	PECAM‐1 heterophilic heparin/glycosaminoglycan binding eliminated	K149, R150 and R151 from domain 2, and E211, H225 and R227 from domain 3, all mutated to alanine
REN‐MP_Y→F_	PECAM‐1 tyrosine phosphorylation eliminated	Y662 and Y685 mutated to phenylalanine

Summarized are descriptions of REN cells expressing wild‐type mouse PECAM‐1, or mutant mouse PECAM‐1 in which ligand binding or tyrosine phosphorylation have been eliminated.

### Functional assessments of the PECAM‐1‐expressing REN cell lines

Three approaches were used to assess the specificity of the targeted mutations and their functional consequences on the activities of the REN cell lines: mixed aggregation studies, immunostaining of confluent monolayers for PECAM‐1 and heparin surface binding.

#### Mixed aggregation studies

**Figure 2:**
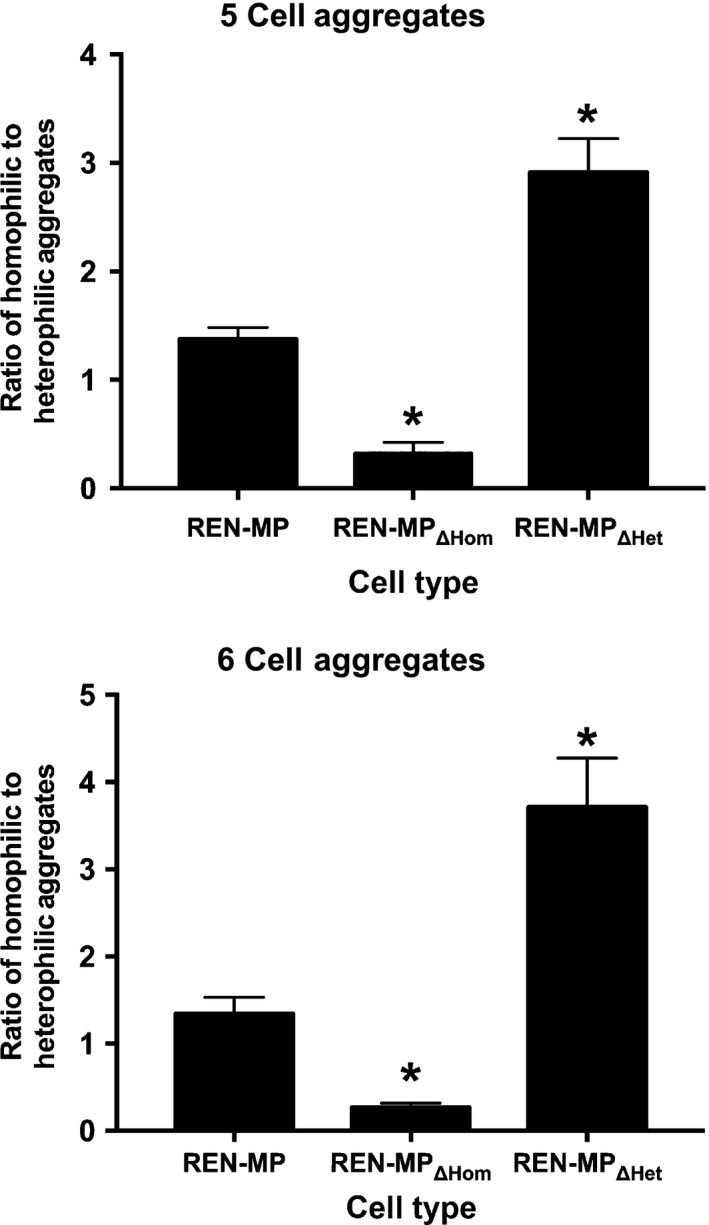
Mixed aggregation studies. Mixed aggregation studies were done in which non‐PECAM‐1 expressing REN cells (non‐fluorescent) were mixed with REN‐MP, REN‐MP_ΔHom_, or REN‐MP_ΔHet_ (all expressing GFP) and the frequency distribution of the various five and six cell aggregates determined, with the data expressed as the ratio of homophilic to heterophilic aggregates. For REN‐MP both homophilic and somewhat less heterophilic aggregates were noted in the mixed aggregation studies, suggesting that both homophilic and heterophilic interactions are present with homophilic binding being the more dominant interaction for REN‐MP. For mixed aggregation studies with REN‐MP_ΔHom_ (homophilic binding disabled) the population of aggregates was depleted of the homophilic aggregates. On the other hand, the population aggregates for the REN‐MP_ΔHet_ cells (heterophilic binding disabled) was enriched (at the expense of the mixed aggregates) with the homophilic aggregates. Values are mean ± SE; *n *= 4–7; *P < 0.05 compared to REN‐MP; each experiment included at least 25 aggregates in the analysis.

A well‐established assay for assessing the homophilic versus heterophilic ligand‐binding properties of a cell adhesion molecule involves mixed aggregation studies in which cells expressing the molecule of interest are mixed with controls cells lacking the molecule (DeLisser et al. 1993, 1994; Sun et al. 1996a). In these so called “mixed aggregation studies”, homophilic interactions result in aggregates that are enriched with cells expressing the molecule of interest, whereas heterophilic binding (to other molecules on surface of the cell) are responsible for aggregates that are composed of both cell types. Therefore to assess the effects on ligand binding of our targeted mutations in the extracellular domain of PECAM‐1 mixed aggregation studies were done in which non‐PECAM‐1 expressing REN cells were mixed with REN‐MP, REN‐MP_ΔHom_, or REN‐MP_ΔHet_ (Fig. 2). Focusing on the 5 and 6 cell aggregates, we determined the frequency distribution of the homophilic versus heterophilic aggregate types for each of the REN cell line. For REN‐MP, both homophilic, with somewhat less heterophilic aggregates were noted in the mixed aggregation studies (ratio of homophilic to heterophilic aggregates~ 1.4), suggesting that both homophilic and heterophilic interactions are present, with homophilic binding being the more dominant interaction for REN‐MP in this assay system. For mixed aggregation studies with REN‐MP_ΔHom_ (homophilic binding disabled) the population of aggregates was depleted of the homophilic aggregates (ratio of homophilic to heterophilic aggregates < 0.33). In contrast, the population aggregates for the REN‐MP_ΔHet_ cells (heterophilic binding disabled) was enriched (at the expense of the heterophilic aggregates) with the homophilic aggregates (ratio of homophilic to heterophilic aggregates >2.9). Together these data confirm the specificity of the mutations in REN‐MP_ΔHom_ and REN‐MP_ΔHet_ in terms of, respectively, disrupting homophilic or heparin/GAG‐mediated heterophilic ligand binding.

#### Concentration of PECAM‐1 in intercellular junctions

**Figure 3:**
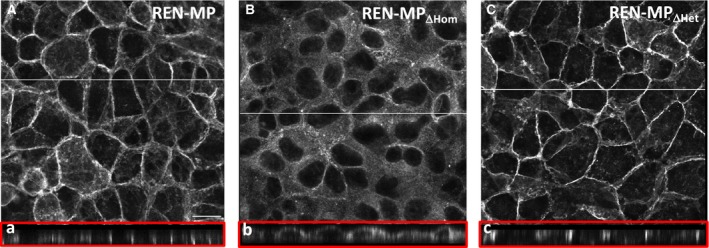
Immunofluorescence staining and confocal imaging of confluent monolayers of REN cells expressing wild‐type or mutant mouse PECAM‐1. Cells were plated and grown to confluence and then subjected to immunofluorescence staining with anti‐PECAM‐1 antibody. Cells were imaged by confocal microscopy (A, B, C) with reconstruction in the *Z*‐axis (a, b, c), along the line indicated in the upper panels, to provide representative cross‐sectional images. Staining of REN‐MP (A, a) and REN‐MP_ΔHet_ (C, c) show localization of PECAM‐1 in intercellular junctions, whereas for REN‐MP_ΔHom_ (B, b) the protein remains diffuse and fails to concentrate at cell–cell junctions.

For a functional assessment of homophilic‐binding activity we determined, by immunostaining, the concentration of PECAM‐1 in the intercellular junctions of confluent cell monolayers (Fig. 3), a process mediated by PECAM‐1‐PECAM‐1 binding interactions between adjacent cells (Sun et al. 2000). Immunofluorescent staining of monolayers of REN‐MP demonstrated the characteristic concentration of PECAM‐1 at the borders of adjacent cells (Fig. 3A). In contrast, for REN‐MP_ΔHom_, PECAM‐1 was distributed diffusely over the surface of the membrane rather than concentrated in intercellular junctions, consistent with a disruption of PECAM‐1‐dependent homophilic binding (Fig. 3B). The pattern of staining for REN‐MP_ΔHet_ was comparable to that of REN‐MP, confirming the preservation PECAM‐1‐dependent homophilic binding in this cell line (Fig. 3C).

#### Heparin cell surface binding

To probe for presence of heparin/GAG binding, the REN cell lines were stained with Cy5.5‐labeled heparin and then analyzed by FACS analysis, identifying the population of cells with high‐affinity binding for heparin (Table 2). For REN‐MP, there was a more than eightfold increase in the number of high‐affinity heparin‐binding cells, compared to control REN cells. The size of the population of these cells for REN‐MP_ΔHom_, was similar to that of REN‐MP, consistent with preservation of PECAM‐1‐dependent, heparin/GAG‐mediated heterophilic binding. However, for REN‐MP_ΔHet_ the numbers high‐affinity heparin‐binding cells were significantly less than that of REN‐MP indicative of a loss of heparin/GAG binding.

**Table 2:** Heparin binding of the PECAM‐1‐expressing REN cell lines

REN cell type	REN‐MP	REN‐MP_ΔHom_	REN‐MP_ΔHet_
High Affinity Heparin Binding (Fold increase over control REN Cells)	8.4 ± 3.7	6.3 ± 2.4	2.4 ± 0.8*

The REN cell lines were stained with Cy5.5‐labeled heparin and then analyzed by FACS analysis, identifying the population of cells with high‐affinity binding for heparin. Data are expressed as fold increase over control REN cells. Values are mean ± SE; *n *= 3; *P < 0.05 compared to REN‐MP; experiments were done in duplicate.

Together these data confirm that REN‐MP_ΔHom_ and REN‐MP_ΔHet_ are functionally distinct. Namely, homophilic binding has been diminished in REN‐MP_ΔHom_ such that PECAM‐1 is no longer able to concentrate in intracellular junctions, whereas for REN‐MP_ΔHet_, heparin/GAG‐dependent, heterophilic binding is sufficiently compromised so that heparin cell surface binding is significantly reduced (relative to REN‐MP).

### Disabling homophilic or heparin/GAG‐dependent heterophilic binding inhibits PECAM‐1‐dependent cell motility

**Figure 4:**
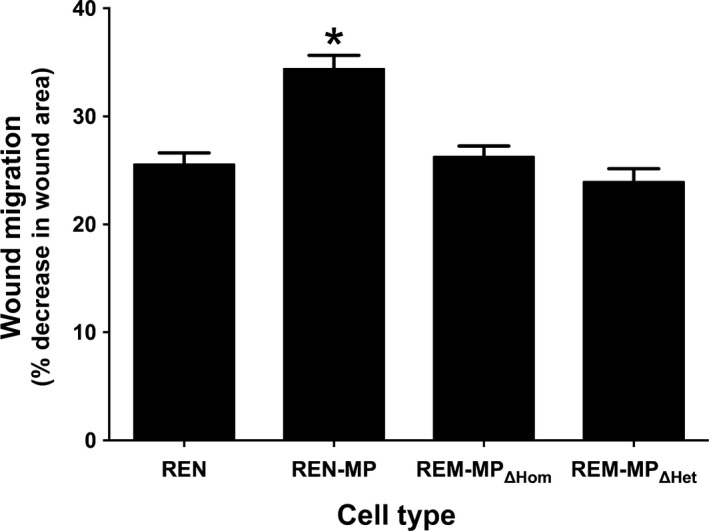
Wound‐induced migration of REN cells expressing wild‐type or mutant mouse PECAM‐1. Linear defects were made in confluent monolayers of REN, REN‐MP, REN‐MP_ΔHom_, or REN‐MP_ΔHet_ and closure of the wound after 24 h was assessed by computer‐assisted image analysis. Data are presented as percent decrease in wound area. Would migration was increased by the expression of mouse PECAM‐1, an effect that was lost if ligand binding was disabled. Data are presented as means ± SE; *n* = 20–30; *P < 0.01 compared to REN cells.

The expression of PECAM‐1 stimulates wound‐induced cell migration of REN cells, an activity that is lost if PECAM‐1 tyrosine phosphorylation is disabled (Cao et al. 2002; O'Brien et al. 2004; Zhu et al. 2010). To assess the role of PECAM‐1‐dependent ligand binding in this process, wound‐induced migration was studied in REN‐MP_ΔHom_ and REN‐MP_ΔHet_ cells (Fig. 4). We observed, consistent with previous reports, that the presence of PECAM‐1 increased REN cell wound‐induced migration by more than 35 percent compared to control REN cells. This augmentation in cell motility, however, was not demonstrated by either REN‐MP_ΔHom_ or REN‐MP_ΔHet_ cells, suggesting that both homophilic and heparin/GAG‐mediated heterophilic ligand interactions of PECAM‐1 play a role in the ability of the molecule to promote cell motility.

### PECAM‐1‐mediated tyrosine phosphorylation and increases in focal adhesion turnover are not inhibited by disruption of ligand binding

**Figure 5:**
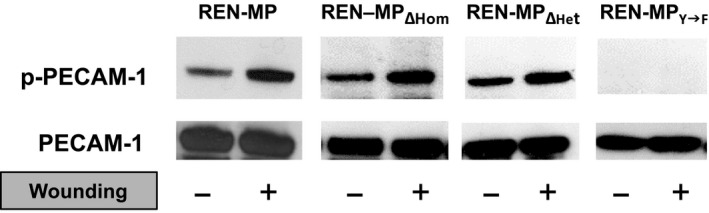
PECAM‐1 tyrosine phosphorylation in REN cells expressing wild‐type or mutant mouse PECAM‐1. Cell lysates from non‐wounded and wounded monolayers of REN‐MP, REN‐MP_ΔHom_, REN‐MP_ΔHet,_ and REN‐MP_Y→F_ cell lines were immunoprecipitated and then immunoblotted with anti‐PECAM‐1 antibody or antiphospho‐tyrosine antibody. For the REN‐MP cells, wounding induced tyrosine phosphorylation, with a similar response observed in the REN‐MP_ΔHom_, and REN‐MP_ΔHet_. As expected, mutation of Y662 and 685 (REN‐MP_Y→F_) resulted in the loss of PECAM‐1 tyrosine phosphorylation.

With respect to the mechanisms by which it promotes cell migration, PECAM‐1 stimulates the turnover of focal adhesions of migrating cells, phenomenon that appears to dependent on PECAM‐1 tyrosine phosphorylation (of residues Y662 and Y685) and the recruitment of the SHP‐2 phosphatase to the cell membrane (O'Brien et al. 2004; Zhu et al. 2010). In studies with the various cell types, the wounding of confluent monolayers of REN‐MP induces tyrosine phosphorylation (Fig. 5). We observed, however, that although wound‐induced, PECAM‐1 tyrosine phosphorylation was lost in REN‐MP_Y→F_, it was preserved in both REN‐MP_ΔHom_ and REN‐MP_ΔHet_, suggesting that these phosphorylation events are not mediated through the ligand interactions of PECAM‐1.

**Figure 6:**
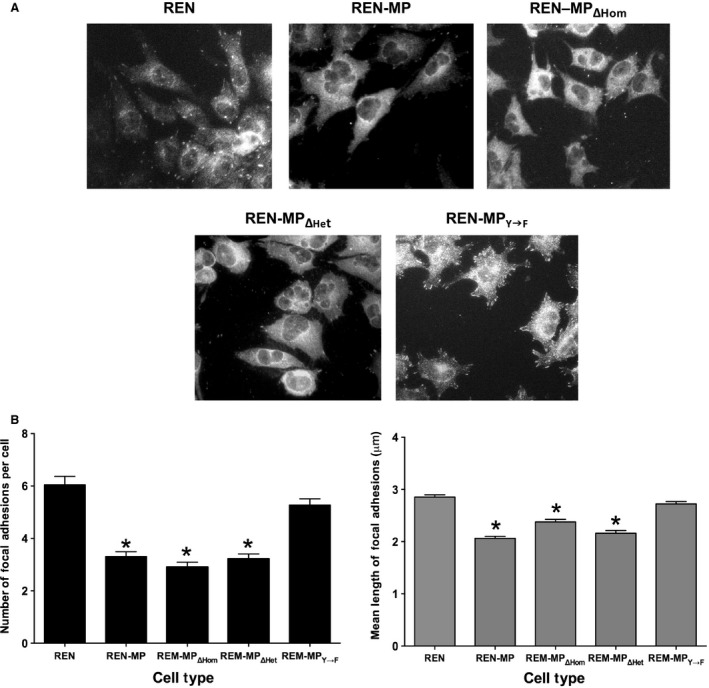
Characterization of focal adhesions in REN cells expressing wild‐type or mutant mouse PECAM‐1. Monolayers in which linear wounds had been placed were stained with antipaxillin antibody to identify focal adhesions (A), which are readily detected in the REN cell and in REN‐MP_Y→F_ mutant, but much less so in the REN‐MP, REN‐MP_ΔHom_, and REN‐MP_ΔHet_ cell lines. For migrating cells the number of focal adhesions per cell (B) and the mean length of focal adhesions (C) were determined. Consistent with increased turnover of the focal adhesions, the number and length of focal adhesions were decreased in REN‐MP, with a similar pattern of response observed in the REN‐MP_ΔHom_, and REN‐MP_ΔHet_. The behavior of REN‐MP_Y→F_ was similar to that of the control REN cells. Data are presented as means ± SE; *n *≥ 150 focal adhesions; *P < 0.0001 compared to REN cells.

Studies assessing the turnover of focal adhesions during wound‐induced migration, phenomenon downstream of PECAM‐1 tyrosine phosphorylation, were subsequently done (Fig. 6). Staining for paxillin (Fig. 6A) confirmed that the number of focal adhesions/cell (Fig. 6B), as previously described (O'Brien et al. 2004), as well as their mean length (Fig. 6B) were reduced in REN‐MP compared to control REN and REN‐MP_Y→F_, cells, consistent with increased, tyrosine phosphorylation‐dependent cycling (assembly/disassembly) of these structures (Wehrle‐Haller 2012). For the other cell types, we observed that these measures of focal adhesion dynamics for REN‐MP_ΔHom_ and REN‐MP_ΔHet_ were similar to that of REN‐MP. Taken together these data indicate that in terms of cell migration, PECAM‐1 tyrosine phosphorylation, and the stimulation of focal adhesion dynamics that is linked to it, are independent of PECAM‐1‐mediated ligand interactions.

### Disabling PECAM‐1‐dependent homophilic or heterophilic binding inhibits PECAM‐1‐mediated extension of filopodia

**Figure 7:**
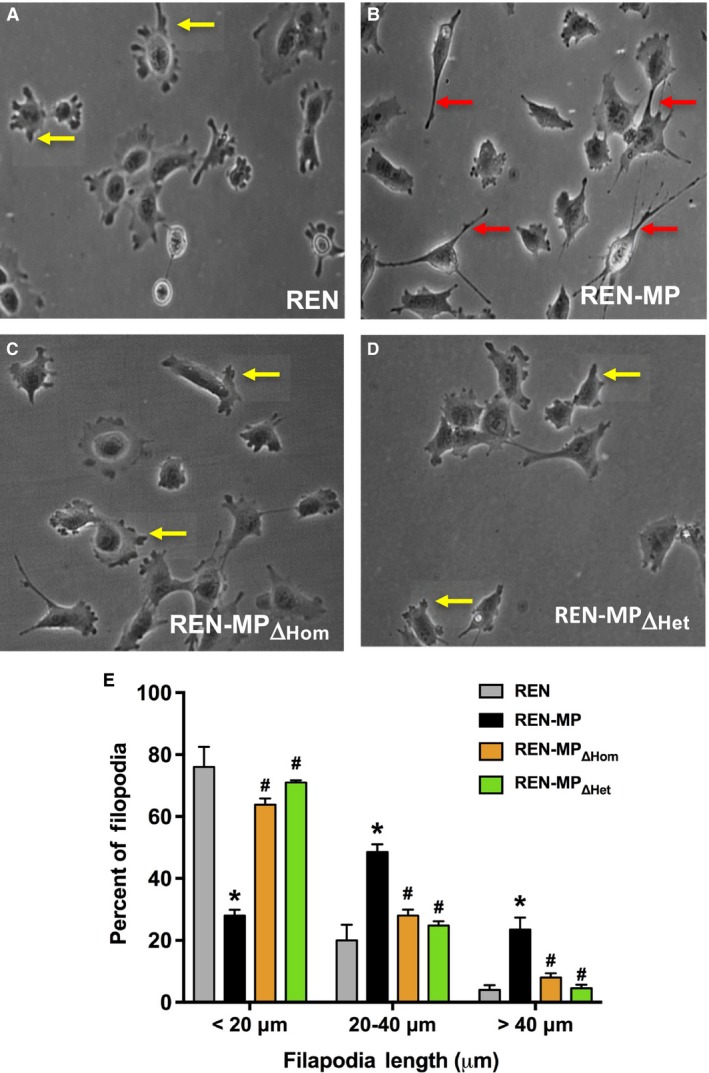
Filopodia formation by REN cells expressing wild‐type or mutant mouse PECAM‐1. Shown are REN (A), REN‐MP (B), REN‐MP_ΔHom_ (C), and REN‐MP_ΔHet,_ (D) cells. The filopodia for the REN‐MP were longer (red arrows) compared to those of the REN cells (yellow arrows). The morphology of the filopodia for the REN‐MP_ΔHom_ and REN‐MP_ΔHet,_ cells were reminiscent of the REN cells. The frequency distribution of the lengths of the filopodia (<20 μm, 20–40 μm, and >40 μm) was plotted (E). The majority of filopodia (>70%) emanating from the REN‐MP cells were greater than 20 μm, frequently extending beyond 40 μm, whereas for the REN‐MP_ΔHom_ and REN‐MP_ΔHet,_ cells, like the REN cells, a large majority (> 60%) of the filopodia were less than 20 μm. Data are presented as means ± SE; *n *= 3–4, *P < 0.0001 compared to REN cells; ^#^ *P *< 0.0001 compared to REN‐MP; for each experiment at least 30 cells and 70 filopodia were included in the analysis.

A second mechanism for the activity of PECAM‐1 in cell motility involves the formation of filopodia (Gupton and Gertler 2007; Mattila and Lappalainen 2008), an effect that is mediated in part by upregulating the expression of Cdc42 (Cao et al. 2009), a Rho GTPase closely associated with the elaboration of these cellular extensions (Install and Machesky 2009; Ridley 2011). Consistent with previous published data (Cao et al. 2009; Zhu et al. 2010), the expression of mouse PECAM‐1 in REN cells resulted in filopodia that were significantly longer than those of control REN cells (Fig. 7). Thus while <25% of the filopodia were >20 μm for the REN cells, in REN‐MP, >70% of the filopodia were >20 μm. In contrast, the frequency distribution for the filopodia length for REN‐MP_ΔHom_, and REN‐MP_ΔHet_, were similar to that of the control REN cells. Significantly, there were no differences in the number of filopodia/cell for the four cell lines (Table 3) suggesting that PECAM‐1 is involved in the extension of filopodia, rather than their initiation, an activity that is mediated by both homophilic and heparin/GAG‐dependent heterophilic PECAM‐1 ligand interactions.

**Table 3:** Number of filopodia/cell

REN cell type	REN control	REN‐MP	REN‐MP_ΔHom_	REN‐MP_ΔHet_
Filopodia/Cell	2.9 ± 0.1	3.1 ± 0.04	2.9 ± 0.1	2.9 ± 0.1

Presented are data on the number of filopodia associated with each cell. Data are presented as means ± SE; *n *= 33–42 images, with >400 filopodia analyzed.

**Figure 8:**
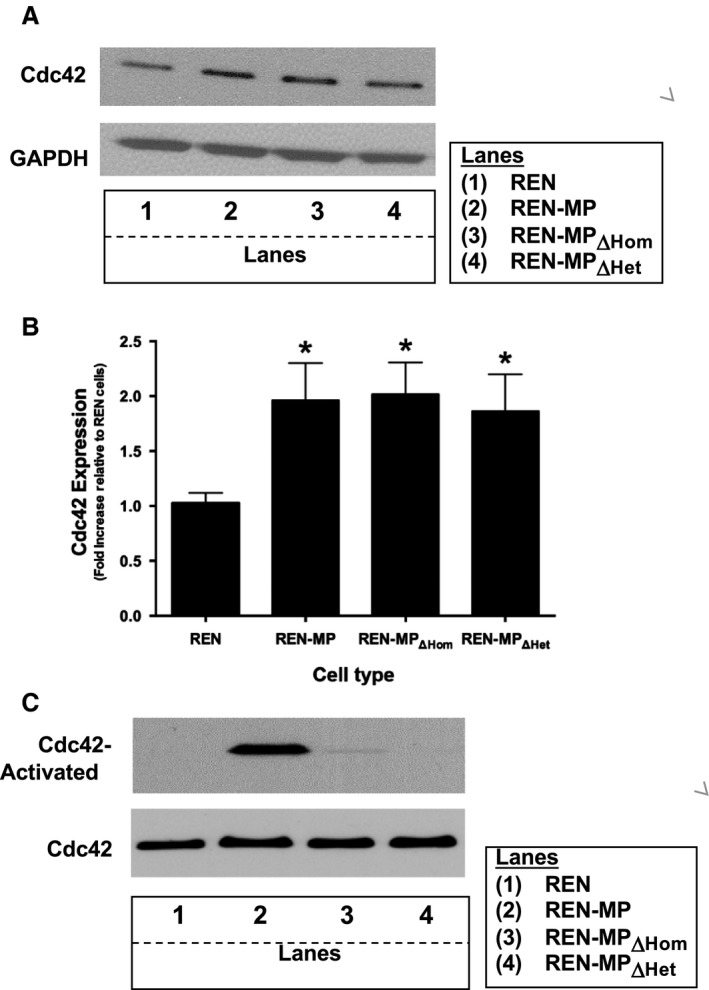
Cdc42 expression and activity in REN cells expressing wild‐type or mutant mouse PECAM‐1. Cell lysates from REN, REN‐MP, REN‐MP_ΔHom_, and REN‐MP_ΔHet,_ cell lines were immunoblotted with anti‐Cdc 42 antibody (A). Cdc42 expression was increased in REN‐MP as well as in REN‐MP_ΔHom_, and REN‐MP_ΔHet_ compared to control REN cells, which was confirmed by densitometric analysis (B). The data were normalized to GPDH and expressed as fold change compared to REN cells. Data are presented as means ± SE (*n *= 3; *P < 0.05, compared to REN cells). Activated Cdc42 was precipitated from cell lysates of subconfluent migrating cells (see Methods) and then detected by Western blot (C). Cdc42 activation was noted to be markedly increased in the REN‐MP cells (lane 2), but was only minimally detected in the REN, REN‐MP_ΔHom_, and REN‐MP_ΔHet,_ cells (lanes1, 3 and 4). These data are representative of three experiments.

To explore these findings further we determined Cdc42 protein level and activity in the various REN cell lines (Fig. 8). Although increased expression of Cdc42 was associated with the presence of PECAM‐1, the suppression of filopodia extension in REN‐MP_ΔHom_ and REN‐MP_ΔHet_ noted above was not associated with a downregulation in the expression of Cdc42 of these cell lines (Fig. 8A and B). In subsequent studies we assessed the Cdc42 activity of subconfluent, motile REN cell types. We observed that while Cdc42 activity was significantly increased in the REN‐MP cells, it was reduced to minimally detectable, control levels in both the REN‐MP_ΔHom_ and REN‐MP_ΔHet_ cell lines (Fig. 8C). These data suggest that PECAM‐1 has two distinct effects on Cdc42 protein level and activity. The expression of PECAM‐1 upregulates the levels of Cdc42 independent of its ligand interactions, whereas in the context of cell motility, PECAM‐1, through both homophilic and heparin/GAG‐mediated heterophilic‐binding interactions, stimulates the activity of Cdc42.

### Concentration of PECAM‐1 in the tips of filopodia is lost after the disruption of PECAM‐1‐dependent homophilic binding

**Figure 9:**
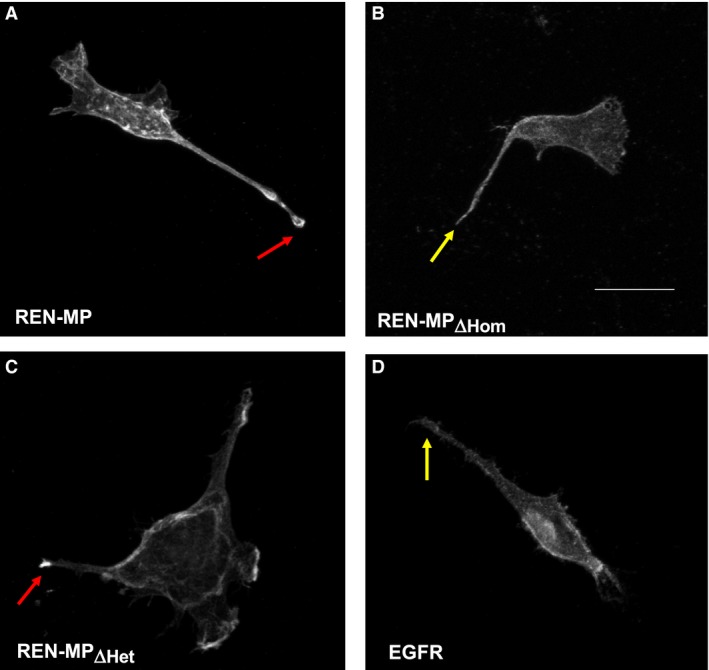
Immunofluorescence staining single, migrating of REN cells expressing wild‐type or mutant mouse PECAM‐1. Subconfluent, migrating REN‐MP (A), REN‐MP_ΔHom_ (B), and REN‐MP_ΔHet,_ (C) cells were immunofluorescently stained for PECAM‐1. Staining of control REN cells for EFGR is shown in panel D. PECAM‐1 was noted to frequently concentrate in the tips of the filopodia extending from REN‐MP and REN‐MP_ΔHet_ cells (yellow arrow), whereas this pattern was infrequently observed for REN‐MP_ΔHom_. (red arrow) For control REN cells, EFGR did not concentrate in filopodial tips. Scale bar (panel B) = 20 μm.

To further investigate the role of PECAM‐1 in the elongation of filopodia, immunostaining for PECAM‐1 was performed on migrating cells, with particular attention to the distribution of PECAM‐1 in filopodia >20 μm. For the REN‐MP cells, PECAM‐1 was frequently concentrated in the tips of filopodia (Fig. 9A), with 41 ± 1% of filopodia demonstrating this pattern of staining. While a similar frequency of 37 ± 1.5% was also observed for the filopodia of the REN‐MP_ΔHet_ cells (Fig. 9C), PECAM‐1 was detected in the tips of only 15 ± 1% of the filopodia extending from the REN‐MP_ΔHom_ (Fig. 9B; *n* = 3 with at least 60 filopodia analyzed for each experiment; *P *<* *0.0006). These findings are unlikely to be artifacts, as staining for the cell surface receptor epidermal growth factor receptor (EGFR) never demonstrated the presence of EGFR in the tips of filopodia (Fig. 9D). These data indicate for actively motile cells that PECAM‐1 concentrates in the tips of filopodia, a process that appears to be mediated by homophilic interactions between PECAM‐1 molecules within the cell membrane.

### Concentration of PECAM‐1 in the tips of filopodia in mouse endothelial cells is inhibited by antibody that blocks homophilic but not heterophilic adhesion

**Figure 10:**
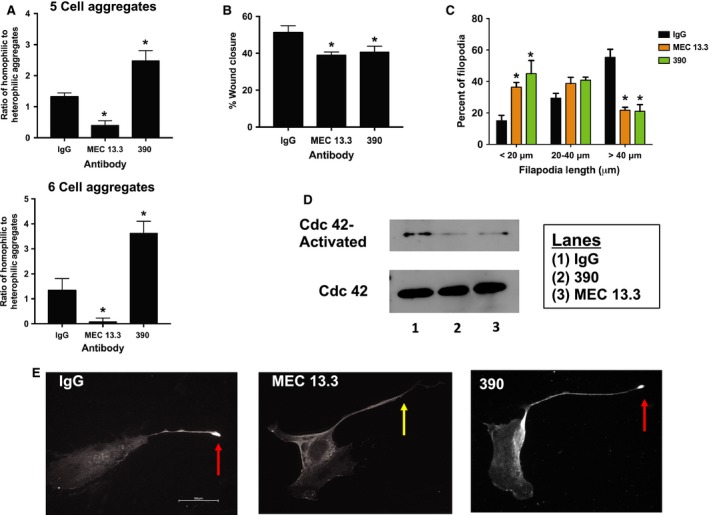
Effects of MEC 13.3 and 390 Antibodies on mouse endothelial cells. Shown are studies of the effects of IgG, MEC 13.3 and 390. For these studies the cells were exposed to 50 μg/ml of IgG or antibody during the duration of the assay (30 min for the mixed aggregation studies and 24 h for the others). (A) Mixed aggregation studies of non‐PECAM‐1 expressing REN cells mixed with REN‐MP were done and the frequency distribution of the various 5 and 6 cell aggregates were determined, with the data expressed as the ratio of homophilic to heterophilic aggregates. Data are presented as means ± SE; *n *= 3–6; *P < 0.05 and <0.01, respectively, for the 5 cell and 6 cell aggregates compared to REN cells; for each experiment at least 25 aggregates were included in the analysis. (B) H5V wound migration with data expressed as percent wound closure. Data are presented as means ± SE; *n *= 3–4; *P < 0.005 compared to IgG; and are representative of three experiments. (C) Frequency distribution of H5V filopodia length, < 20 μm versus 20–40 μm versus >40 μm. Data are presented as means ± SE; *n *= 5; *P < 0.05 and 0.0001 compared to IgG, respectively, for <20 μm and >40 μm data; for each experiment at least 100 filopodia were analyzed. (D) H5V Cdc42 activation, with the data presented representative of three independent experiments. (E) Immunofluorescence staining for PECAM‐1 of primary mouse ECs illustrating that while PECAM‐1 was frequently observed in the tips of extended filopodia of cells treated with IgG or 390 (red arrow), this was much less frequently noted in the MEC 13.3 treated cells (yellow arrow). Scale bar = 50 μm

To confirm the findings from our studies with the REN cell lines, mouse endothelial cells were treated with the anti‐mouse PECAM‐1 antibodies, MEC 13.3 and 390. These antibodies were employed because of previous studies indicating they have differential effects on PECAM‐1‐dependent adhesion (Chacko et al. 2012). This was confirmed to be the case as mixed aggregation studies with REN‐MP cells demonstrated that the population of homophilic aggregates was diminished by MEC 13.3, whereas 390 enriched the population of homophilic aggregates (at the expense of heterophilic aggregates) (Fig. 10A). The activities of MEC 13.3 and 390 replicated, respectively, the activity REN‐MP_ΔHom_ and REN‐MP_ΔHet_ in mixed aggregation studies (Fig. 2) and thus confirm the specificity of MEC 13. 3 and 390 in, respectively, blocking PECAM‐1‐dependent homophilic and heterophilic adhesion.

We subsequently determined that both antibodies inhibited H5V mouse EC migration (Cao et al. 2009) and filopodia extension (Fig. 10B and C). We further observed that while Cdc42 activity was significantly decreased by either antibody (Fig. 10D), MEC 13.3 (homophilic blocking) but not 390 (heterophilic blocking) suppressed the concentration of PECAM‐1 in the tips of filopodia (Fig. 10E) of primary mouse ECs. Thus while PECAM‐1 was frequently detected in the tips of extended filopodia of cells treated with IgG or 390 (30 ± 0.3 and 25 ± 1, %of filopodia, respectively), this was much less frequently noted in the MEC 13.3 treated cells (10 ± 1% of filopodia; *n* = 3, *P* < 0.0001 compared to IgG or 390; for each experiment at least 35 filopodia >20 μm were analyzed). These data with mouse ECs confirm the findings from the REN cell lines and provide further evidence for the differential involvement of PECAM‐1‐ligand interactions in PECAM‐1‐dependent motility and the extension of filopodia.

## Discussion

To define the involvement of PECAM‐1 ligand binding in PECAM‐1‐dependent cell migration, we performed studies with REN cells expressing wild‐type mouse PECAM‐1 or mouse PECAM‐1 in which either homophilic or heparin/GAG‐mediated heterophilic ligand binding had been disabled. We found that PECAM‐1‐dependent cell migration was inhibited by disruption of either PECAM‐1‐mediated homophilic or heparin/GAG‐dependent heterophilic ligand interactions. This inhibition in cell motility was independent of PECAM‐1 tyrosine phosphorylation or changes in the dynamics of focal adhesions. Instead, we observed that disabling homophilic or heparin/GAG‐mediated heterophilic ligand binding of PECAM‐1 inhibited the extension of filopodia and the activation of Cdc42. We also found that PECAM‐1 concentrated at the tips of extended filopodia, an effect that was mediated through homophilic interactions. Importantly, similar patterns of activities were observed in studies of murine ECs treated with antibodies that were confirmed to be specific in their blocking of mouse PECAM‐1 homophilic or heterophilic adhesion.

A variety of model systems have implicated a role for PECAM‐1 in in vivo angiogenesis (DeLisser et al. 1997, 2006; Zhou et al. 1999; Cao et al. 2002, 2009; Solowiej et al. 2003; Dimaio et al. 2008), an activity mediated through an ability to stimulate or enhance endothelial cell motility (Cao et al. 2002, 2009; O'Brien et al. 2004; Kondo et al. 2007; Garnacho et al. 2008; Zhu et al. 2010). In terms of understanding the mechanistic, molecular basis for this activity, studies to date have focused on the role of PECAM‐1‐dependent signaling. These studies suggest that PECAM‐1 tyrosine phosphorylation mediates the recruitment and the activation of the tyrosine phosphatase SHP‐2, which in turn promotes the turnover of focal adhesions as well as the extension of filopodia, phenomena that are integral to cell motility (Gupton and Gertler 2007; Mattila and Lappalainen 2008).

Filopodia are one of several plasma membrane protrusions observed at the leading edge of migrating cells (Gupton and Gertler 2007; Mattila and Lappalainen 2008; Ridley 2011). Composed of tight parallel bundles of polymerized actin, these finger‐like projections contain adhesion molecules that are able to bind to the extracellular matrix and/or initiate intercellular signaling (Install and Machesky 2009; Bornschlog 2013; Blanchoin et al. 2014). These structural and molecular features are consistent with their activity in probing and sensing the surroundings of migrating cells and in facilitating directed cell movement. During angiogenesis, numerous filopodia emanate from endothelial cells at the tips of angiogenic sprouts, where they facilitate rapid and persistent EC migration as well as vessel anastomosis (Gerhardt et al. 2003; Phng et al. 2013). Although VEGF activation has been implicated in the formation endothelial tip cell filopodia, our understanding of the elaboration of filopodia in ECs remains incomplete (De Smet et al. 2009; Siekman et al. 2013).

As noted above, we have previously shown that the presence of PECAM‐1 also promotes the extension of filopodial protrusions in ECs and PECAM‐1‐expressing cells through processes that involve tyrosine phosphorylation of its cytoplasmic domain (Cao et al. 2009; Zhu et al. 2010). In this report we extend those findings by directly demonstrating that both homophilic and heparin/GAG‐dependent heterophilic PECAM‐1 ligand binding are involved (Fig. 7 and 10C). However, unlike tyrosine phosphorylation of the cytoplasmic domain, PECAM‐1‐dependent ligand interactions are not required for PECAM‐1‐mediated increases in the turnover of focal adhesions (Fig. 6), the other established mechanism by which PECAM‐1 stimulates cell migration (O'Brien et al. 2004).

Cdc42, a small GTPase of the Rho superfamily, has long been established as an important signaling molecule in the formation of filopodia (Gupton and Gertler 2007; Mattila and Lappalainen 2008; Install and Machesky 2009; Ridley 2011). Targets of Cdc42 include stimulators of actin polymerization such as WASP and N‐WASP nucleation‐promoting factors; the formin mDia2, which induces the formation of unbranched actin filaments; and insulin‐receptor substrate p53 (IRSp53), a regulator of anticapping proteins. In a previous study, we reported that the expression of PECAM‐1 increased Cdc42 levels (Cao et al. 2009), consistent with its activity in promoting the formation of filopodia. We now show that in addition to upregulating the expression of Cdc42, PECAM‐1, independent of changes in the level of Cdc42, also promotes the activation of Cdc42, an activity that requires both homophilic as well as heparin/GAG‐dependent heterophilic ligand binding (Figs. 8 and 10D). As noted previously there are a number of Cdc42 targets involved in the formation of filopodia and thus studies are ongoing in REN cell surrogates and in mouse ECs to determine which of them might be regulated by PECAM‐1.

However, while both homophilic and heterophilic ligand interactions are involved in the extension of filopodia and in the activation of Cdc42, they are not functionally redundant. This is indicated by our data on the location of PECAM‐1 within filopodia. Specifically, PECAM‐1 was observed to frequently concentrate in the tips of filopodia, a pattern of localization that was dependent on homophilic, but not heparin/GAG‐dependent heterophilic binding (Figs. 9 and 10E). This concentration of PECAM‐1 in filopodial tips is consistent with previous reports demonstrating that VEGFR‐2 and VEGFR‐3, two proangiogenic surface receptors, are present in the filopodial extensions of endothelial tip cells (Gerhardt et al. 2003; Tammela et al. 2008).

On the basis of the data presented in this report, we offer the following working hypothesis as a guide for future studies on the potential involvement of PECAM‐1‐dependent ligand binding in the extension of filopodia. As the filopodia begins to emanate from the cell, homophilic interactions between PECAM‐1 molecules in the cell membrane lead to clustering of the molecule (Duke and Graham 2009; Caré and Soula 2011), particularly at the tips of extending filopodia. PECAM‐1 within the membrane of the filopodia is not freely diffusible to the rest of the cell surface and thus its surface density in the filopodia is likely to be high, conditions that might favor these cis‐homophilic interactions (Sun et al. 1996a). Clustering of PECAM‐1 subsequently enables high‐affinity heterophilic binding to proteoglycans in the matrix that may be reminiscent of the affinity modulation observed with clustered integrins (Maheshwari et al. 2000) or CD44 (Sleeman et al. 1996). Engagement of proteoglycans by PECAM‐1 then transduces signals that activate Cdc42 and thus promote the extension of filopodia. Studies are underway to further investigate this hypothesis. In terms of EC function during angiogenesis, we note that this model does not preclude roles for PECAM‐1‐ligand interactions at the tips of filopodia that go beyond triggering processes that stimulate actin polymerization. Thus the PECAM‐1‐matrix interactions might be adhesive, serving to pull the cell forward or anchor/stabilize the cell as it moves (Maheshwari et al. 2000) and/or PECAM‐1‐PECAM‐1 interactions between molecules on the filopodia of adjacent tip cells might facilitate the anastomosis of angiogenic sprouts (Phng et al. 2013).

## Conflicts of Interest

The authors have no conflicts of interest to declare.
